# Hypercholesterolemia Diagnosis, Treatment Patterns, and 12-Month Target Achievement in Clinical Practice in Germany in Patients with Familial Hypercholesterolemia

**DOI:** 10.3390/jcm11133810

**Published:** 2022-06-30

**Authors:** Anselm K. Gitt, Ulrich Laufs, Winfried März, W. Dieter Paar, Peter Bramlage, Nikolaus Marx, Klaus G. Parhofer

**Affiliations:** 1 Medical Clinic B, Department of Cardiology, Herzzentrum Ludwigshafen, Bremserstr. 79, 67117 Ludwigshafen, Germany; gitta@klilu.de; 2 Klinik und Poliklinik für Kardiologie, University of Leipzig Medical Center, Liebigstr. 20, 04103 Leipzig, Germany; ulrich.laufs@medizin.uni-leipzig.de; 3 Synlab Academy, 68163 Mannheim, Germany; winfried.maerz@synlab.com; 4 Medical Clinic V, Medical Faculty Mannheim, University of Heidelberg, Theodor-Kutzer-Ufer 1–3, 68167 Mannheim, Germany; 5 Clinical Institute of Medical and Chemical Laboratory Diagnostics, University of Graz, Auenbruggerplatz 15, 8036 Graz, Austria; 6 Medical Department, Sanofi-Aventis Deutschland GmbH, Lützowstr. 107, 10785 Berlin, Germany; dieter.paar@sanofi.com; 7 Institute for Pharmacology and Preventive Medicine, Bahnhofstr. 20, 49661 Cloppenburg, Germany; 8 Clinic for Cardiology, Angiology and Intensive Medicine, University Hospital Aachen, Pauwelsstr. 30, 52074 Aachen, Germany; nmarx@ukaachen.de; 9 Medical Clinic IV—Großhadern, Ludwig Maximilian University of Munich, Marchioninistr. 15, 81377 Munich, Germany; klaus.parhofer@med.uni-muenchen.de

**Keywords:** Familial hypercholesterolemia, Germany, lipid levels, lipid-lowering therapy

## Abstract

Background: Familial hypercholesterolemia (FH) is a highly prevalent disorder and a risk factor for early coronary artery disease. The objective of this registry was to document the clinical characteristics of patients with definite FH in Germany and to document lipid profiles, lipid-lowering therapy, and lipid target achievement during longitudinal follow-up. Methods: HYDRA-FH was a national, prospective, multicenter, non-interventional registry conducted in 35 centers in Germany. Consecutive adult patients with definite FH were included (*n* = 241). Results: In the cross-sectional analysis (*n* = 233), lipid-lowering therapy involved statins (82.0%), ezetimibe (31.8%), and PCSK9 antibodies (18.5%); 11.2% of patients were receiving no lipid-lowering drugs. Median lipid levels were: low-density lipoprotein cholesterol (LDL-C) 134 mg/dL (3.5 mmol/L), high-density lipoprotein cholesterol (HDL-C) 48 mg/dL (1.2 mmol/L), triglycerides 160 mg/dL (1.9 mmol/L), total cholesterol 211 mg/dL (5.5 mmol/L). Values were above the normal threshold (150 mg/dL) for LDL-C in 72.9%, total cholesterol in 29.7%, and triglycerides in 45.0% of patients. After the 12-month follow-up (*n* = 145), only 17.2% had LDL-C < 70 mg/dL, and 20.7% had either LDL-C < 70 mg/dL or a reduction of ≥50% versus baseline. Conclusion: This study provides insight into the clinical characteristics and current treatment status of patients with FH in Germany. Many patients with FH do not achieve recommended lipid levels.

## 1. Introduction

Familial hypercholesterolemia (FH) is a highly prevalent genetic dyslipidemia, affecting 1 in 250 people worldwide, [1] and is a significant risk factor for early coronary artery disease (CAD) [2,3]. However, it is generally underdiagnosed and undertreated in routine clinical practice [4,5,6,7]. In Europe, the median age at diagnosis of FH is 44.4 years with a prevalence of coronary disease of 17.4% that increases with untreated low-density lipoprotein cholesterol (LDL-C) levels [8]. The European Society of Cardiology (ESC) and European Atherosclerosis Society (EAS) guidelines [9,10] emphasize the importance of early diagnosis of heterozygous FH in order to identify patients at high risk of subsequent cardiovascular disease and who will benefit from the prompt initiation of lipid-lowering therapy [11].

High-intensity statin treatment is generally recommended as first-line pharmacotherapy for patients with FH, with combination therapy implemented if lipid targets are not reached [12]. PCSK9 antibodies are recommended for very-high-risk FH patients who do not achieve lipid goals on statins plus ezetimibe [10,12]. However, many patients with FH do not achieve the recommended target levels for LDL-C [5,13,14,15].

Few reports on the status of patients with FH in Germany have been published [16,17,18]. The prevalence rate has been estimated to be between 1 in 278 (based on the Dutch Lipid Clinic Score) and 1 in 295 (US-MEDPED score) [19]. A report from a German registry (CaReHigh) in 2018 indicated that insufficient treatment was common and less than 20% of patients achieved LDL-C targets [17]. To gain insights into the current treatment situation for patients with FH in Germany, the multicenter HYDRA-FH registry study was initiated. The aim was to document the clinical characteristics of patients with definite FH seen in clinical practice in Germany (cross-sectional study). In addition, lipid profiles, lipid-lowering therapy, and lipid target achievement were documented during the longitudinal follow-up.

## 2. Materials and Methods

HYDRA-FH was a national, prospective, multicenter, non-interventional registry study conducted in 35 centers in Germany. Study sites were distributed across the country to ensure the data gathered were representative of patients treated with FH throughout Germany. The study was performed in accordance with the ethical principles outlined in the Declaration of Helsinki and all applicable amendments laid down by the World Medical Assemblies and the International Conference Harmonization guidelines for Good Clinical Practice. The study protocol was approved by the “Landesärztekammer Rheinland-Pfalz” under the number 837.391.16 (10710). All patients provided written informed consent prior to documentation.

### 2.1. Site Selection and Patient Selection

Sites that routinely treat patients with definite FH were selected in order to represent the clinical care routine for this patient group in Germany as realistically as possible.

Patient inclusion criteria were limited to the presence of definite (diagnosed) FH (Dutch Lipid Score [20], Simon Broome Criteria [21]) in adult patients (≥18 years) and the requirement for written informed consent from the patient. Apart from concurrent participation in a clinical trial, no explicit exclusion criteria were mandated.

Suitable patients were enrolled consecutively at the study sites. Within the framework of this registry, all therapies that were approved for the treatment of this patient group could be applied. Treatment was solely at the discretion of the treating physician; the study did not provide explicit treatment guidelines.

### 2.2. Sample Size

We assumed that we would be able to recruit a total of 500 patients in 100 participating centers. Assuming a 50% target achievement rate, this would result in a precision of ±4.4% (95% confidence interval (CI) 45.6 to 54.4). Furthermore, we determined that 500 patients would allow us to report adverse events with a 95% probability for an event that occurs in 6/1000 patients, e.g., 3 times in a 500-patient study.

We estimated a necessary recruitment duration of 12 months, but as the recruitment was far off the intended number of 500 patients, we decided to discontinue recruitment after the inclusion of 241 patients. This changed the potential precision of the 50% target achievement rate determination to ±6.2% (95% CI 43.8 to 56.2) and decreased the number of patients with adverse events detectable to 14 in 1000.

### 2.3. Data Collection

This analysis reports the results of a baseline visit and one follow-up visit at 12 ± 4 months. Patient data were documented in electronic case report forms (eCRF). Adverse events were reported from the time the physician obtained the patient’s informed consent until 7 days after the end of the observation period. Quality control measures included on-site visits at selected study centers (>5% of centers), including source data verification using the interview technique.

Due to the fact that data were collected in 35 centers, including office-based physicians, hospitals, and medical care centers, the determination of lipid values was not standardized. The observational nature of this study prevented us from using a centralized lab.

### 2.4. Objectives

The first objective was to document the clinical characteristics of patients with definite FH seen in clinical practice in Germany in a cross-sectional study. The second objective was to document lipid profiles, lipid-lowering therapy, and lipid target achievement during the longitudinal follow-up.

Lipid parameters included LDL-C, high-density lipoprotein cholesterol (HDL-C), total cholesterol, and triglycerides. Lipid target achievement was assessed based on the reduction in LDL-C from baseline to the 12-month follow-up, including absolute and relative reductions. Absolute and relative changes in total cholesterol, HDL-C, and triglycerides from baseline to 12 months were also assessed.

Lipid-lowering therapies, including non-drug lipid lowering therapies (lifestyle and nutrition) and lipid-lowering pharmacotherapy used by patients as well as the percentage of patients using such therapies at baseline and at the 12-month follow-up were recorded. Drugs used for secondary cardiovascular prevention and the percentage of patients receiving such pharmacotherapy at baseline and the 12-month follow-up were also recorded. Drugs were captured in classes rather than specific substances. No frequency was documented.

Information on cardiovascular and cerebrovascular events was also obtained, including the percentage of patients with complications at baseline and the percentage with non-fatal events that occurred between baseline and the 12-month follow-up.

### 2.5. Statistics

**Figure 1:**
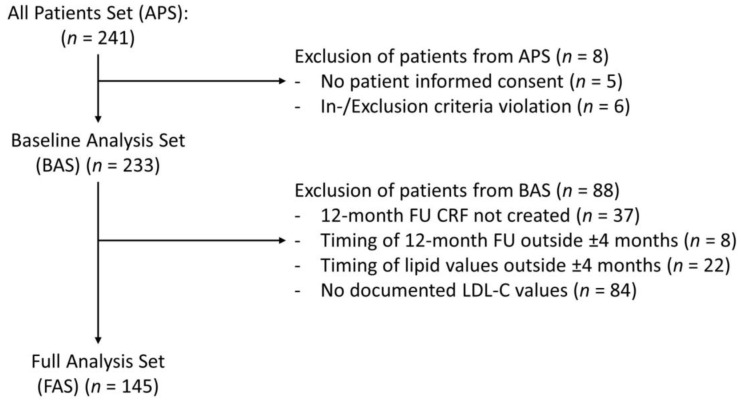
Patient flow. APS, all patients set; BAS, baseline analysis set; FAS, full analysis set; CRF, case report form; FU, follow-up; LDL-C, low density lipoprotein cholesterol.

All collected parameters were evaluated descriptively. Two sets of patients were defined for the analyses: (1) the Baseline Analysis Set (BAS; *n* = 233) was defined to capture all patient characteristics and events irrespective of the follow-up duration. This was to capture adverse events in patients receiving a specific drug which would then be discontinued early. The BAS consisted of all patients who met all inclusion criteria according to the observation plan and at least one further documented entry in the first documentation section of the eCRF. It was used for the evaluation of cross-sectional data. (2) The Full-Analysis Set (FAS; *n* = 145) was defined to describe the follow-up of patients within the first 12 months as the follow-up was incomplete (see Figure 1 for reasons). It consisted of all BAS patients with documentation of LDL-C values, lipid-lowering therapy at the time of an index event, and the 12-month follow-up data. The FAS was used for the evaluation of longitudinal data.

Categorical variables were presented as absolute and relative frequencies. Continuous variables were presented as absolute number (*n*), mean ± standard deviation, or median with interquartile range (IQR). In the FAS, the McNemar test was used for comparing threshold values at baseline and the 12-month follow-up, whereas the Friedman test was used for assessing changes between baseline and the 12-month follow-up. No imputations were made to replace missing data. For time-to-event analyses, patient data were censored based on the last known follow-up value (i.e., in the case of premature drop-out, the last existing value was used).

All statistical analyses were carried out using the SAS^®^ package (version 9.4, Cary, NC, USA).

## 3. Results

A total of 241 patients was recruited by 32 office-based physicians, 2 hospitals, and 1 medical care center. Sites recruited an average of 6.2 ± 6.2 patients during the total recruitment period of 143.6 ± 118.9 days. Among 241 recruited patients, 233 fulfilled all inclusion and exclusion criteria and were included in the BAS, of whom 145 were included in the FAS (Figure 1).

### 3.1. Cross-Sectional Analysis (BAS, n = 233)

Patients had a median age of 61 years, 58.8% were male, and the median body mass index was 28.5 kg/m^2^ (Table 1). The most frequent phenotypic findings were xanthelasma in 48 patients (21.1%) and xanthomas in 28 patients (12.3%). A functional mutation in low density lipoprotein receptor (LDLR), apolipoprotein B (apoB), or PCSK9 genes was documented in only 21 of 99 patients (21.2%) who underwent testing. Most patients had a family history of elevated cholesterol levels (*n* = 140, 60.1%), coronary heart disease (*n* = 125, 53.7%), and myocardial infarction (*n* = 114, 48.9%). The most frequent comorbid disease conditions seen in patients included coronary heart disease (*n* = 126, 55.3%), a history of percutaneous coronary intervention (*n* = 78, 34.2%), and heart failure (*n* = 52, 22.8%).

Treatment at baseline was largely based on statins (*n* = 191, 82.0%), ezetimibe (*n* = 74, 31.8%), and PCSK9 antibodies (*n* = 43, 18.5%) (Table 2). Overall, 114 (48.9%) were receiving monotherapy (98 statins, 5 PCSK9 antibodies), and 93 (39.9%) were receiving combination therapy, while 26 patients (11.2%) were receiving no lipid-lowering drugs. Cardiovascular pharmacotherapy was dominated by antihypertensive drugs, followed by platelet aggregation inhibitors and antidiabetic drugs (Table 3).

Median lipid levels values were: LDL-C 134 mg/dL (3.5 mmol/L), HDL-C 48 mg/dL (1.2 mmol/L), triglycerides 160 mg/dL (1.8 mmol/L), and total cholesterol 211 mg/dL (5.5 mmol/L) (Table 4). Values were above the normal threshold for LDL-C (threshold 150 mg/dL) in 94 patients (72.9%), total cholesterol (threshold 260 mg/dL) in 65 patients (29.7%), and triglycerides (threshold 172 mg/dL) in 98 patients (45.0%).

### 3.2. Longitudinal Analysis at 12 Months (FAS, n = 145)

Of the 233 patients in the BAS, 88 were excluded from the longitudinal analysis, resulting in 145 patients being available for the longitudinal analysis (FAS) (Figure 1). Reasons for exclusion included a lack of follow-up, follow-up or lipid value measurements being performed outside the time window of 12 ± 4 months, or absence of documented LDL-C values. Mean follow-up in the FAS was 379 ± 28 days.

At baseline, patients included in the FAS had similar mean age, body mass index, and gender distribution compared to those excluded from the FAS, but they had a higher rate of phenotypic findings, more often a family history, and more comorbid cardiovascular disease conditions (Table 1). FAS patients were further characterized by more intense lipid-lowering and cardiovascular pharmacotherapy, whereas differences in non-drug supportive lifestyle habits were negligible (Table 2 and Table 3). LDL-C, total cholesterol, and HDL-C values all tended to be lower at baseline than in the cross-sectional population (BAS).

**Figure 2:**
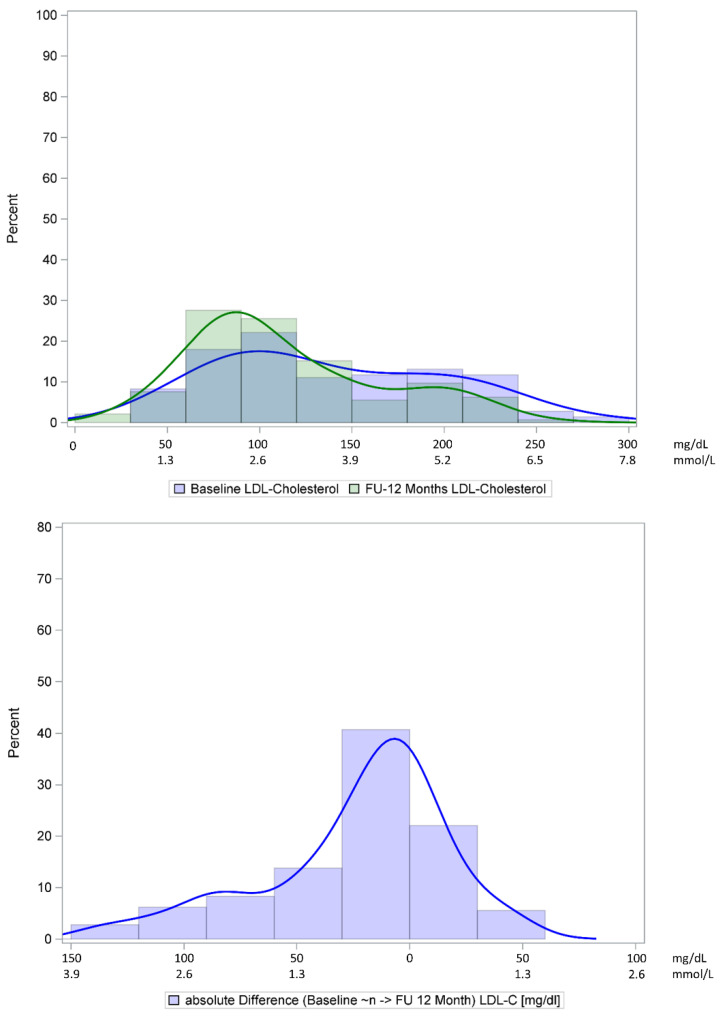
Distribution of LDL-C values (mg/dL) at 12 months (FAS). FAS, full analysis set; FU, follow-up; LDL-C, low-density lipoprotein cholesterol.

Drug and non-drug lipid lowering strategies changed only slightly during the 12 months of follow-up. At 12 months, fewer patients received statins, fibrates or ezetimibe, while slightly more patients received PCSK9 antibodies (Supplementary Figure S1). Physical exercise increased (23.5% vs. 15.9% exercising 2–3 times per week) and more patients were non-smokers (73.8% vs. 67.6%). After 12 months, LDL-C decreased by a median of 14 mg/dL. Figure 2 illustrates the changes in the lipid profile of the FAS population over 12 months. At 12 months, 17.2% achieved an LDL-C of <70 mg/dL and 20.7% had either LDL-C < 70 mg/dL or a reduction of ≥50% versus baseline. Total cholesterol decreased by a median of 15 mg/dL to a final value of 188 mg/dL (4.9 mmol/L) and triglycerides decreased by a median of 8 mg/dL to a final median value of 155 mg/dL (1.8 mmol/L).

### 3.3. Outcomes Analysis

Outcome data were collected at 12 months in the FAS (Table 5). No patients died. Non-fatal events that were reported included two patients (1.4%) with myocardial infarction, three (2.1%) who underwent a percutaneous coronary intervention, and four (2.8%) who were hospitalized (for a median duration of 3 days).

## 4. Discussion

This study provides insights into the clinical characteristics and current treatment situation of patients with FH in Germany. Most patients received lipid-lowering pharmacotherapy, but target LDL-C levels were achieved in only a minority of patients. 

### 4.1. Patient Characteristics

Patients generally had typical characteristics associated with FH, including cardiovascular comorbidities and a family history of CAD. The proportion of patients with a family history of elevated cholesterol levels was similar to that reported for the German CaReHigh registry (60%) and although cardiovascular disease was categorized differently in the two studies, the overall picture of a strong family history was consistent [17]. The proportion of patients with comorbid coronary heart disease was somewhat higher in our study (55%) compared with the CaReHigh registry (35%), but similar to the level generally reported across other countries [9]. Almost one fourth of patients in our study had a history of heart failure, which is mainly caused by coronary heart disease. A study involving patients with FH in Norway demonstrated a doubling of the risk of hospitalization for heart failure in patients with FH compared to the general population [22]. Heart valve disease was prevalent in 15.9% of patients, which is substantially lower compared with previously reported prevalence in this patient group [23,24,25], but higher than in the general population [26]. Almost three-quarters of patients had an LDL-C level above the normal threshold.

### 4.2. Genetic Testing

Amongst patients for whom genetic testing results were available, only 21% had a mutation affecting the LDLR, apoB, or PCSK9 genes, which is lower than the rates of 69–77% reported previously in German patients with probable/definitive FH [27] and closer to the rates of 23–35% reported for patients with suspected/possible FH [27,28]. In our study, genetic testing results were available for less than half of the study population. In Germany, genetic testing for FH is performed at the discretion of the treating physician and our results suggest a reluctance to order such tests to confirm the diagnosis. This has also been reported for the CaReHigh Registry, which found that only 11% of FH patients underwent genetic testing [17]. Testing may be important because there is evidence that the availability of a molecular diagnosis of FH can lead to an increase in the intensity of medication that patients are prescribed [13].

### 4.3. Treatment

Most patients in our study were receiving lipid-lowering pharmacotherapy based on statins. The next most frequent drugs were cholesterol absorption inhibitor and PCSK9 antibodies. These drug classes are consistent with those recommended by the guidelines for treatment of FH [10,12]. Almost half of patients were receiving monotherapy (generally a statin). Although statin therapy remains the first pharmacological approach for the management of FH, the majority of patients do not achieve the recommended LDL-C level targets with statins alone [29]. Notably, 11% of patients were receiving no lipid-lowering medication. An even higher rate of 27% was reported for the CaReHigh registry [17]. The researchers suggested an underestimation of the patients’ vascular risk by treating physicians or the fear of potential drug side effects, such as statin-associated muscle symptoms, to be the major reasons for not receiving any lipid-lowering therapy, which may also be the case in our study [17]. In addition, current guidelines state that lipid-lowering medications should not be administered during pregnancy and the period of breastfeeding due to lack of concrete data on possible side effects [12]. Although most patients in our study had adopted some lifestyle and/or dietary modifications, intensive pharmacotherapy is recommended for patients with FH in order to reduce the risk of CAD [10,12]. Current evidence suggests that combining dietary modifications and exercise with drug therapy may be more efficient in reducing the lipid profile, yet there is still a need for further research [29,30,31]. A further analysis of the combined treatment approach was not possible in the current study due to an insufficient number of patients to provide each subgroup with enough statistical power, especially for describing it in the context of treatment decisions and lipid values achieved.

Overall, the data in our study are consistent with insufficient treatment of FH, which is known to be a problem globally [5,6,7].

### 4.4. Longitudinal Follow-Up

Some studies have found an increase in the level of lipid-lowering therapy over time in patients with FH [13,32]. Follow-up of patients in our study found only slight changes in lipid-lowering strategies after 12 months with a small increase in the number of patients receiving a PCSK9 antibody. This would be consistent with recommendations to use a PCSK9 antibody in very-high-risk patients who have not achieved target lipid levels on a statin plus ezetimibe [10]. It is possible that greater increases in the intensity of therapy would be seen after a longer follow-up of patients in the HYDRA-FH registry.

Target LDL-C levels were not achieved in most patients. After 12 months, less than one-fifth of patients had achieved an LDL-C level <70 mg/d. This is consistent with the findings of the CaReHigh registry, which found that 18% of FH patients with CVD and 15% of those without CVD were at or below target levels of 70 mg/dL and 100 mg/dL, respectively, despite lipid-lowering therapy [17]. Studies from other countries have reported similar or even lower levels of target attainment on conventional lipid-lowering therapy [13,14,15,32], although there is evidence that prescription of PCSK9 antibodies can help improve the control of LDL-C levels [13]. 

### 4.5. Limitations

The current study has several limitations associated with its non-interventional design in a routine practice setting. Centers may not have been representative for the clinical care of these patients in general practice, as they were specialized in the care of these patients. Information was not available for all parameters for some patients; most notably, results for genetic testing were available for less than half of patients. However, the main limitation of the study was the high loss to follow-up, which restricted the number of patients who could be included in the longitudinal analysis. The most common reasons were a lack of documented LDL-C values during the 12-month follow-up period or a lack of any follow-up data. The reasons for these absences were not recorded. 

## 5. Conclusions

This study provides insights into the clinical characteristics and current treatment pattern of patients with FH in Germany. Patients generally have typical characteristics associated with FH, including cardiovascular comorbidities and a family history of CAD. Only less than half of patients in our study had available genetic testing results, indicating low overall genetic diagnosis rates. Even with lipid-lowering treatment, most patients do not achieve recommended LDL-C levels. The results highlight the need for improved strategies for the management of patients with FH in Germany. More effective treatment options for dyslipidemia are needed.

## Figures and Tables

**Table 1:** Patient characteristics for the BAS and FAS.

	BAS *n* = 233	FAS *n* = 145
Age, years mean ± SD	61.1 ± 14.0 (*n* = 233)	60.6 ± 13.7 (*n* = 145)
Median (IQR)	62 (53, 71)	63 (55, 70)
Male gender, *n* (%)	137/233 (58.8)	82/145 (56.6)
Body mass index, kg/m^2^ mean ± SD	28.5 ± 4.50 (*n* = 228)	28.2 ± 4.3 (*n* = 145)
Median (IQR)	28 (25, 31)	28 (25, 31)
Phenotypic findings—no. of participants		
Xanthelasma, *n*/N (%)	48/227 (21.1)	38/145 (26.2)
Xanthomas, *n*/N (%)	28/228 (12.3)	18/145 (12.4)
Arcus cornealis, *n*/N (%)	20/227 (8.8)	15/145 (10.3)
Funct. mutation in LDLR, apoB or PCSK9 gene, *n*/N (%)	21/99 (21.2)	15/57 (26.3)
Age at initial diagnosis, years, mean ± SD	46.4 ± 10.5 (*n* = 20)	48.1 ± 9.6 (*n* = 15)
Median (IQR)	47 (41, 55)	49 (43, 58)
Family history—no. of participants	(*n* = 228)	(*n* = 145)
Family history of elevated cholesterol levels, *n* (%)	140 (60.1)	97 (66.9)
Family history of coronary heart disease, *n* (%)	125 (53.7)	87 (60.0)
Family history of myocardial infarction, *n* (%)	114 (48.9)	81 (55.9)
Family history of cerebrovascular disease, *n* (%)	53 (22.8)	37 (25.5)
Family history of tendon xanthomas, *n* (%)	19 (8.2)	13 (9.0)
Family history of arcus cornealis, *n* (%)	8 (3.4)	3 (2.1)
Lipoprotein apheresis therapy, *n* (%)	11 (4.8) *	9 (6.2)
Cardiovascular history—no. of participants	(*n* = 228)	(*n* = 145)
Coronary heart disease, *n* (%)	126 (55.3)	89 (61.4)
Percutaneous coronary intervention, *n* (%)	78 (34.2)	57 (39.3)
Acute coronary syndrome, *n* (%)	46 (20.2)	32 (22.1)
Aortocoronary bypass, *n* (%)	30 (13.2)	22 (15.2)
Stroke, *n* (%)	12 (5.3) **	9 (6.2)
Transitory ischemic attack, *n* (%)	3 (1.3)	1 (0.7)
Comorbidities—no. of participants	(*n* = 228)	(*n* = 145)
Heart failure, *n* (%)	52 (22.8)	38 (26.2)
Heart valve disease, *n* (%)	37 (15.9)	25 (17.2)
Renal insufficiency, *n* (%)	24 (10.5)	17 (11.7)
Stable angina pectoris, *n* (%)	22 (9.6)	18 (12.4)
Peripheral arterial occlusive disease, *n* (%)	17 (7.5)	11 (7.6)
Atrial fibrillation, *n* (%)	12 (6.6)	9 (6.2)
Carcinoma, *n* (%)	11 (4.8)	6 (4.1)

Legend: BAS, baseline analysis set; FAS, full analysis set; SD, standard deviation; IQR, interquartile range; LDLR, low density lipoprotein receptor; apoB, apolipoprotein B; percentages are calculated based on available data; * missing for 5 patients vs. 233, ** missing for 1 patient vs. 228.

**Table 2:** Lipid-lowering therapy in the BAS and FAS.

	BAS Baseline	FAS Baseline	FAS 12-Month FU	*p*-Value
	(*n* = 233)	(*n* = 145)	(*n* = 145)	FAS 12 vs. FAS
Fibrates, *n* (%)	5 (2.2)	2 (1.4)	1 (0.7)	0.3173
Ezetimibe, *n* (%)	74 (31.8)	55 (37.9)	51 (35.2)	0.0153
PCSK9 antibody, *n* (%)	43 (18.5)	34 (23.4)	36 (24.8)	0.5637
Statin, *n* (%)	191 (82.0)	125 (86.2)	117 (80.7)	<0.0001
Other lipid-lowering therapy, *n* (%)	22 (9.4)	14 (9.7)	14 (9.7)	0.0679
None, *n* (%)	26 (11.2)	11 (7.6)	6 (4.1)	1.000
Physical exercise *	(*n* = 233)	(*n* = 145)	(*n* = 145)	<0.0001
None, *n* (%)	99 (42.5)	57 (39.3)	53 (36.6)	
1× per week, *n* (%)	70 (30.0)	55 (37.9)	47 (32.4)	
2–3× per week, *n* (%)	35 (15.0)	23 (15.9)	34 (23.5)	
>3× per week, *n* (%)	23 (9.9)	10 (6.9)	10 (6.9)	
Fruit and vegetable consumption *	(*n* = 233)	(*n* = 145)	(*n* = 145)	0.3991
≥3× per week, *n* (%)	146 (62.7)	91 (62.8)	86 (59.3)	
<3× per week, *n* (%)	75 (32.2)	50 (34.5)	52 (35.9)	
None, *n* (%)	6 (2.6)	4 (2.8)	6 (4.2)	
Fish consumption *	(*n* = 233)	(*n* = 145)	(*n* = 145)	0.3092
≥2× per week, *n* (%)	49 (21.0)	29 (20.0)	30 (20.7)	
<2× per week, *n* (%)	149 (64.0)	99 (68.3)	90 (62.1)	
None, *n* (%)	29 (12.5)	17 (11.7)	25 (17.2)	
Alcohol consumption (number of alcoholic beverages) *	(*n* = 233)	(*n* = 145)	(*n* = 145)	0.0779
≥2× per day, *n* (%)	24 (10.3)	15 (10.3)	15 (10.3)	
<2× per day, *n* (%)	103 (44.2)	65 (44.8)	70 (48.3)	
None, *n* (%)	101 (43.4)	65 (44.8)	59 (40.7)	
Smoking status *	(*n* = 233)	(*n* = 145)	(*n* = 145)	0.0609
Non-smoker, *n* (%)	153 (65.7)	98 (67.6)	107 (73.8)	
Current smoker, *n* (%)	23 (9.9)	13 (9.0)	11 (7.6)	
Ex-smoker, *n* (%)	51 (21.9)	34 (23.5)	27 (18.6) **	

Legend: BAS, baseline analysis set; FAS, full analysis set; FU, follow-up; * any mismatch in numbers is due to missing data; ** some ex-smokers may have been coded as non-smokers at 12 months; percentages are calculated based on available data.

**Table 3:** Cardiovascular pharmacotherapy in the BAS and FAS.

	BAS Baseline (*n* = 233)	FAS Baseline (*n* = 145)	FAS 12-Month FU (*n* = 145)
P2Y12 antagonist, *n* (%)	18 (7.7)	13 (9.0)	7 (4.8)
Other platelet aggregation inhibitors, *n* (%)	94 (40.3)	62 (42.8)	72 (49.7)
Vitamin-K antagonist, *n* (%)	6 (2.6)	6 (4.2)	3 (2.1)
Direct oral anticoagulant, *n* (%)	7 (3.0)	5 (3.5)	5 (3.5)
Beta blocker, *n* (%)	65 (27.9)	37 (25.5)	53 (36.6)
Angiotensin II receptor blocker, *n* (%)	58 (24.9)	46 (31.7)	47 (32.4)
ACE inhibitor, *n* (%)	84 (36.1)	47 (32.4)	46 (31.7)
Diuretic, *n* (%)	47 (20.2)	29 (20.0)	24 (16.6)
If channel inhibitor, *n* (%)	12 (5.2)	9 (6.2)	3 (2.1)
Calcium channel blocker, *n* (%)	52 (22.3)	38 (26.2)	44 (30.3)
Oral antidiabetic, *n* (%)	55 (23.6)	37 (25.5)	30 (20.7)
GLP-1 receptor agonist, *n* (%)	12 (5.2)	11 (7.6)	10 (6.9)
Insulin, *n* (%)	32 (13.7)	17 (11.7)	15 (10.3)
Anti-angina drug, *n* (%)	6 (2.6)	5 (3.5)	4 (2.8)

Legend: ACE, angiotensin-converting enzyme; BAS, baseline analysis set; FAS, full analysis set; FU, follow-up; GLP-1, glucagon-like peptide-1; information was available for all patients at baseline (BAS, FAS) and FU (FAS).

**Table 4:** Lipid values in the BAS and FAS and change in lipid values in the FAS.

	BAS (*n* = 233)	FAS (*n* = 145)				
	Baseline Median (IQR)	Baseline Median (IQR)	12 Months Median (IQR)	*p*-Value 12 Months vs. BL	Abs. Δ BL—12 Months	Rel. Δ BL—12 Months
**LDL-C, mg/dL**	134 (92, 194)	128 (89, 194)	99 (78, 144)	<0.0001	−14 (−43, 1)	−10 (−33, 2)
**mmol/L**	3.5 (2.4, 5.0)	3.3 (2.3, 5.0)	2.6 (2.0, 3.7)			
>150 mg/dL, *n* (%)	94/219 (72.9)	59 (40.7)	32/145 (22.1)	<0.0001	−18.6	-
<70 mg/dL, *n* (%)	24/219 (11.0)	15/145 (10.3)	25/145 (17.2)	<0.0001	+6.9	-
<70 mg/dL OR 50% red.	-	-	30/145 (20.7)			
**Total cholesterol, mg/dL**	211 (169, 267)	204 (167, 267)	188 (144, 216)	<0.0001	−15 (−48, −1)	−6 (−23, −0)
**mmol/L**	5.5 (4.4, 6.9)	5.3 (4.3, 6.9)	4.9 (3.7, 5.6)			
>260 mg/dL, *n* (%)	65/219 (29.7)	42/143 (29.4)	26/141 (18.4)	<0.0001		
**HDL-C, mg/dL**	48 (40, 59)	47 (40, 59)	46 (41, 55)	0.3266	0 (−4, 2)	0 (−8, 5)
**mmol/L**	1.2 (1.0, 1.5)	1.2 (1.0, 1.5)	1.2 (1.1, 1.4)			
m < 45/f < 55 mg/dL, *n* (%)	112/212 (52.8)	77/140 (55.0)	79/141 (56.0)	0.3778		
**Triglycerides, mg/dL**	160 (114, 214)	165 (115, 211)	155 (101, 206)	0.0027	−8 (−31, 20)	−4 (−21, 19)
**mmol/L**	1.8 (1.3, 2.4)	1.9 (1.3, 2.4)	1.8 (1.1, 2.3)			
>172 mg/dL, *n* (%)	98/218 (45.0)	68/144 (47.2)	58/133 (43.6)	0.0124		

Legend: Abs. Δ, absolute change; BL, baseline; BAS, baseline analysis set; FAS, full analysis set; f, female; IQR, interquartile range; HDL-C, high-density lipoprotein cholesterol; LDL-C, low-density lipoprotein cholesterol; m, male; red., reduction; Rel. Δ, relative change; values are medians (IQR).

**Table 5:** Non-fatal outcomes at 12 months in the FAS.

	FAS at 12 Months (*n* = 145)
Myocardial infarction, *n* (%) [95% CI]	2 (1.4) [0.4–4.9]
Cardiac catheter without PCI, *n* (%)	0 (0.0)
Percutaneous coronary intervention, *n* (%) [95% CI]	3 (2.1) [0.7–5.9]
Bypass surgery, *n* (%)	0 (0.0)
Stroke, *n* (%)	0 (0.0)
Transitory ischemic attack, *n* (%)	0 (0.0)
Hospitalization due to event, *n* (%) [95% CI]	4 (2.8) [1.1–6.9]
Duration of hospitalization due to event, days	
mean ± SD	4.5 ± 3.7
median (IQR)	3 (3, 7)
Rehabilitation, *n* (%)	0 (0.0)
Other inpatient stay, *n* (%) [95% CI]	3 (2.1) [0.7–5.9]
Duration of other inpatient stay, days	
mean ± SD	9.3 ± 8.1
median (IQR)	8 (2, 18)

Legend: CI, confidence interval; FAS, full analysis set; PCI, percutaneous coronary intervention; SD, standard deviation; IQR, interquartile range. Events in the FAS are based on either yes/no or no information. For the purpose of this analysis, it was assumed that all patients with no information (*n* = 138) but with a follow-up visit had not suffered from any of these events.

## Data Availability

Aggregated data are available upon reasonable request from the sponsor.

## Supplementary Materials

The following supporting information can be downloaded at: https://www.mdpi.com/article/10.3390/jcm11133810/s1, Figure S1: Distribution of lipid-lowering medication at baseline and 12 months (FAS).
