# Supplementary material for: Hypercholesterolemia Diagnosis, Treatment Patterns, and 12-Month Target Achievement in Clinical Practice in Germany in Patients with Familial Hypercholesterolemia
Source: J Clin Med. 2022 Jun 30;11(13):3810. doi: 10.3390/jcm11133810 (PMC9267207; doi:10.3390/jcm11133810)
Supplement: Supplementary file 1 [file jcm-11-03810-s001.zip › jcm-1776140-supplementary.pdf]

SUPPLEMENT

Figure S1: Distribution of lipid-lowering medication at baseline and 12 months (FAS).

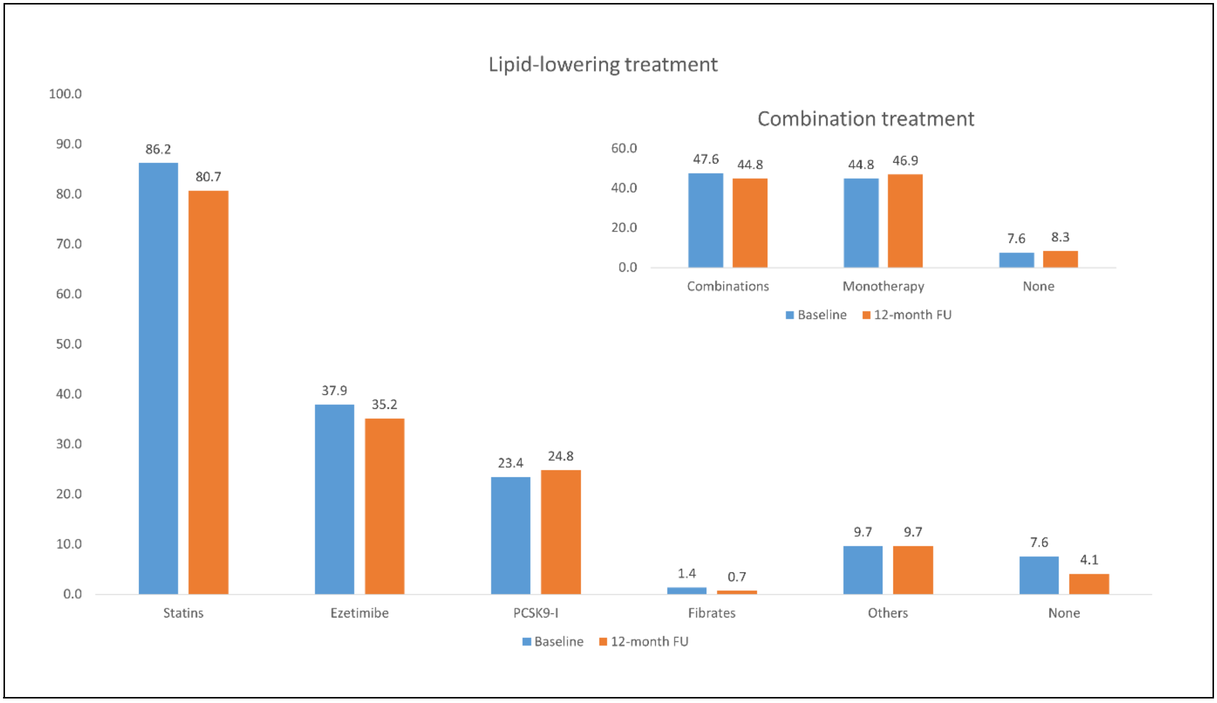

Legend: FAS, full analysis set; FU, follow-up; PCSK9-I, PCSK9 antibody.
